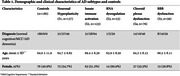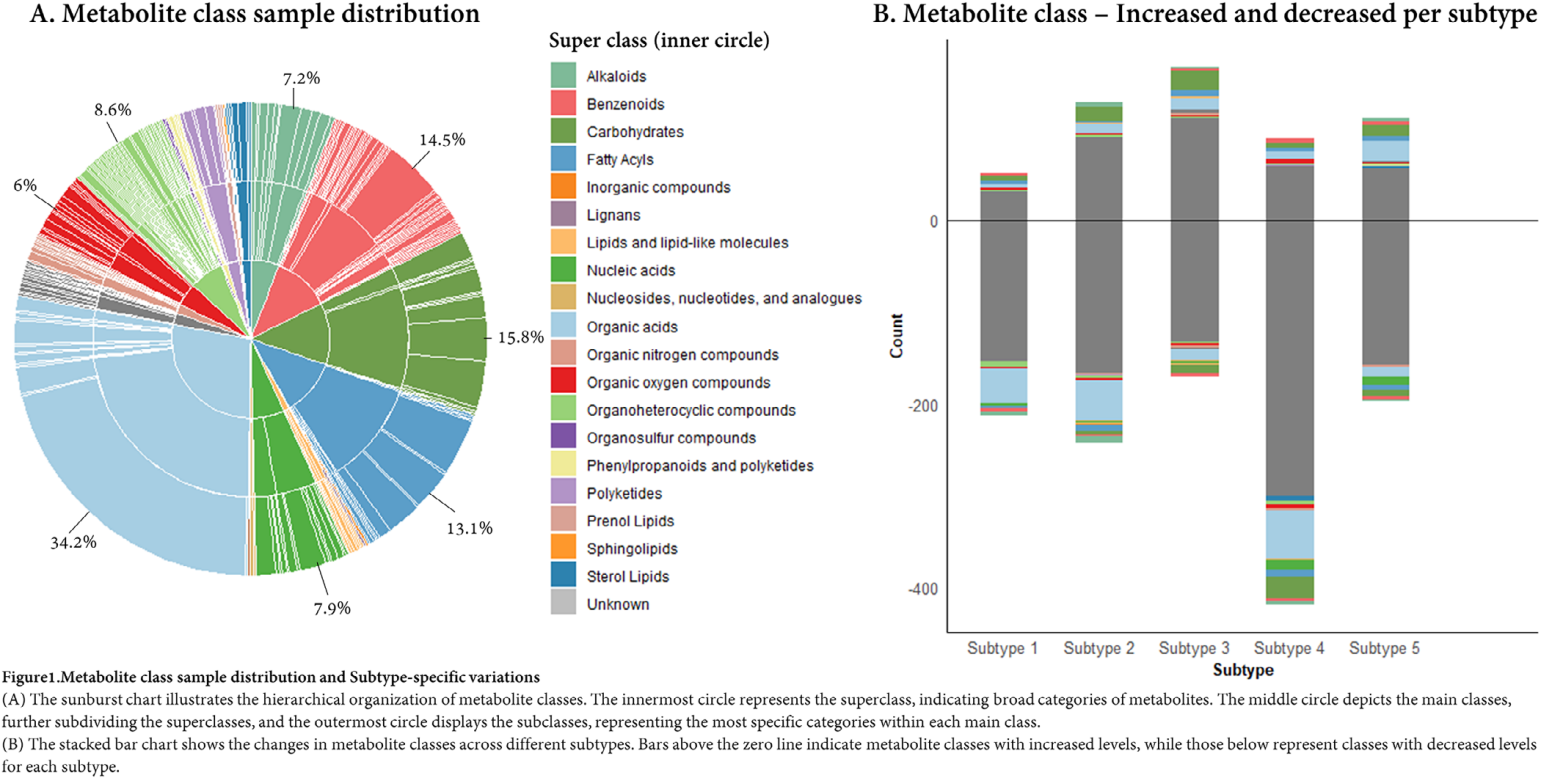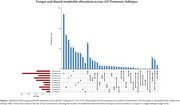# CSF metabolomic signatures across five proteomic subtypes in patients with Alzheimer's disease

**DOI:** 10.1002/alz70855_101527

**Published:** 2025-12-23

**Authors:** Georgia Malliou, Lianne M. Reus, Yolande A.L. Pijnenburg, Wiesje M. van der Flier, Charlotte E. Teunissen, Pieter Jelle Visser, Roel Ophoff, Betty M. Tijms

**Affiliations:** ^1^ Amsterdam Neuroscience, Neurodegeneration, Amsterdam, Netherlands; ^2^ Alzheimer Center Amsterdam, Neurology, Vrije Universiteit Amsterdam, Amsterdam UMC location VUmc, Amsterdam, Netherlands; ^3^ Amsterdam Neuroscience, Neurodegeneration, Amsterdam, North Holland, Netherlands; ^4^ Center for Neurobehavioral Genetics, Semel Institute for Neuroscience and Human Behavior, David Geffen School of Medicine, University of California, Los Angeles, Los Angeles, CA, USA, Los Angeles, CA, USA; ^5^ Amsterdam Neuroscience, Neurodegeneration, Amsterdam, Noord‐Holland, Netherlands; ^6^ Department of Epidemiology and Data Science, Vrije Universiteit Amsterdam, Amsterdam UMC, Amsterdam, North Holland, Netherlands; ^7^ Alzheimer Center, Department of Neurology, Amsterdam UMC, Vrije Universiteit Amsterdam, Amsterdam Neuroscience, Amsterdam, Netherlands; ^8^ Neurochemistry Laboratory, Amsterdam Neuroscience, Program Neurodegeneration, Amsterdam UMC, Vrije Universiteit Amsterdam, Amsterdam, Noord‐Holland, Netherlands; ^9^ Alzheimer Center and Department of Neurology, Amsterdam Neuroscience Campus, VU University Medical Center, Amsterdam, North Holland, Netherlands; ^10^ Alzheimer Center Limburg, School for Mental Health and Neuroscience (MHeNs), Maastricht University, Maastricht, Limburg, Netherlands; ^11^ Amsterdam Neuroscience, Vrije Universiteit Amsterdam, Amsterdam UMC, Amsterdam, North Holland, Netherlands; ^12^ Department of Neurobiology, Care Sciences and Society, Division of Neurogeriatrics, Karolinska Institutet, Stockholm, Stockholm, solna, Sweden; ^13^ Center for Neurobehavioral Genetics, University of California Los Angeles, Los Angeles, CA, USA; ^14^ Alzheimer Center Amsterdam, Neurology, Vrije Universiteit Amsterdam, Amsterdam UMC location VUmc, Amsterdam, North Holland, Netherlands

## Abstract

**Background:**

Alzheimer's disease (AD) is marked by molecular heterogeneity. We previously identified five distinct molecular AD subtypes based on CSF proteomics: neuronal hyperplasticity (S1), innate immune activation (S2), RNA dysregulation (S3), choroid plexus dysfunction (S4), and blood–brain barrier impairment (S5). These subtypes differ in proteins involved in metabolic processes, such as PKM and PYGL (S3) and ATP1A1 (S4), which suggests these subtypes also differ in affected metabolic processes. We investigated whether AD subtypes were associated with specific CSF metabolic signatures.

**Method:**

We conducted untargeted metabolomics on the same CSF samples from 601 individuals in the Amsterdam Dementia Cohort, previously included in our CSF proteomic study (*n* = 416 AD, 185 controls|normal cognition/AD biomarkers; Table 1). Using HILIC‐QTOF (metabolites) and GC‐TOF (biogenic amines) platforms, we detected 2,011 metabolites, with 544 mapped to known classes. Metabolite levels were compared between subtypes and controls using linear regression models adjusted for age and sex (R v4.2.1). Metabolites with statistically different levels (*p* <0.05) underwent pathway enrichment analysis using MetaboAnalyst6.0.

**Result:**

We found 993 metabolites with altered CSF levels between any of the AD subtypes compared to controls. The choroid plexus exhibited the most changes (S4:473, 47.6%) followed by innate immune activation (S2:339, 34.1%), RNA dysregulation (S3:315, 31.7%), blood‐brain barrier (S5:287, 28,9%), and neuronal hyperplasticity (S1:242, 24.4%). These metabolites were mostly mapped to organic acids, carbohydrates, fatty acyls and alkaloids (Figure 1A). Organic acids were increased in S3/S5 but decreased in S1/S2/S4. Carbohydrates were elevated in S1/S2/S3/S5 but mainly reduced in S4 (Figure 1B). Fatty acyls increased in S1/S3/S5 and decreased in S4, while alkaloids increased in S3 and decreased in S1 (Figure 1B). While most metabolites were unique to each subtype (Figure 2), we observed common metabolites that were altered in the decreased categories of S4/S5, predominantly comprising organic acids and carbohydrates. Pathway analysis revealed enrichment for processes including compound trasport (S2/S4/S5), amino acid metabolism (S3/S1), tRNA aminoacylation (S2/S1), and glucose homeostasis (S3).

**Conclusion:**

AD proteomic subtypes exhibit different CSF metabolomic alterations, reflecting heterogeneous pathophysiological mechanisms. This profiling could inform future subtype‐specific therapies targeting metabolism. For example, therapies modulating glucose metabolism, typically tested in general AD populations, may be most effective for patients in S3.